# Vibrational coherence transfer in an electronically decoupled molecular dyad

**DOI:** 10.1038/srep09368

**Published:** 2015-03-23

**Authors:** Felix Schweighöfer, Lars Dworak, Markus Braun, Marc Zastrow, Jan Wahl, Irene Burghardt, Karola Rück-Braun, Josef Wachtveitl

**Affiliations:** 1 Institute of Physical and Theoretical Chemistry, Goethe-University Frankfurt, Max-von-Laue Str. 7, D 60438 Frankfurt/M., Germany; 2 TU-Berlin, Strasse des 17. Juni 135, D 10623 Berlin, Germany

## Abstract

The ring opening of a dithienylethene photoswitch incorporated in a bridged boron-dipyrromethene - dithienylethene molecular dyad was investigated with ultrafast spectroscopy. Coherent vibrations in the electronic ground state of the boron-dipyrromethene are triggered after selective photoexcitation of the closed dithienylethene indicating vibrational coupling although the two moieties are electronically isolated. A distribution of short-lived modes and a long-lived mode at 143 cm^−1^ are observed. Analysis of the theoretical frequency spectrum indicates two modes at 97 cm^−1^ and 147 cm^−1^ which strongly modulate the electronic transition energy. Both modes exhibit a characteristic displacement of the bridge suggesting that the mechanical momentum of the initial geometry change after photoexcitation of the dithienylethene is transduced to the boron-dipyrromethene. The relaxation to the dithienylethene electronic ground state is accompanied by significant heat dissipation into the surrounding medium. In the investigated dyad, the boron-dipyrromethene acts as probe for the ultrafast photophysical processes in the dithienylethene.

The development of femtosecond time resolved spectroscopy with pulses shorter than the period of molecular vibrations has opened a way to monitor coherent vibrational wavepackets in the electronic ground and excited states and added decisive information about the primary processes on the excited and ground state potential energy surfaces of various systems. In this context the correlation between the initial nuclear motion out of the Franck-Condon region and the photochemical reaction is a central issue. For example, the torsional dynamics in the de-/planarization of molecular excited states^1^, the reactive motion in molecular switches^2^ and artificial light-harvesting systems^3^, the charge migration induced lattice vibrations in semiconductor quantum dots^4^ as well as the nuclear motions in metal complexes^5^ have been characterized by coherent wavepackets observed in time domain experiments with femtosecond time resolution. Molecular wavepackets are of particular interest in the coherent photoisomerization of chromophores, a process in which the excitation energy is funneled to certain reactive molecular modes^2^.

The present study describes the ring opening dynamics of the molecular switch dithienylethene (DTE) which is covalently linked to a boron-dipyrromethene (BODIPY) via a molecular bridge (cf. Fig. 1(a)). In femtosecond time resolved absorption measurements the closed DTE was photoexcited selectively. While the electronic excitation is strictly located on the DTE and the bridge, the BODIPY acts as indicator for nuclear motions within the complete molecular dyad.

A distribution of strongly damped low frequency modes, observed at the spectral position of the BODIPY ground state absorption, decays on the sub-picosecond time scale. Additionally, a long lived mode at 143 cm^−1^ is observed. To clarify the underlying photophysics of the molecular dyad, the theoretical frequency spectrum has been calculated. Subsequently, the modes were analyzed regarding their influence on the electronic transition energies. Dominant modes at 97 cm^−1^ and 147 cm^−1^ were found, which exhibit significant geometry changes in the DTE residue and a large displacement of the bridge indicating that the initial geometry change in the DTE is transduced onto the BODIPY. In terms of bond angles the bridge is rigid and therefore acts as a molecular piston. Consequently, the observed vibrational coherence is not induced by the photoexcitation itself but by the mechanical momentum transduction onto the BODIPY via the bridge. Derivative-like transient absorption features at the spectral position of the BODIPY ground state absorption indicate that the fast relaxation to the molecular electronic ground state is accompanied by significant heating of the surrounding medium.

## Results

### Steady state spectroscopy

**Figure 1:**
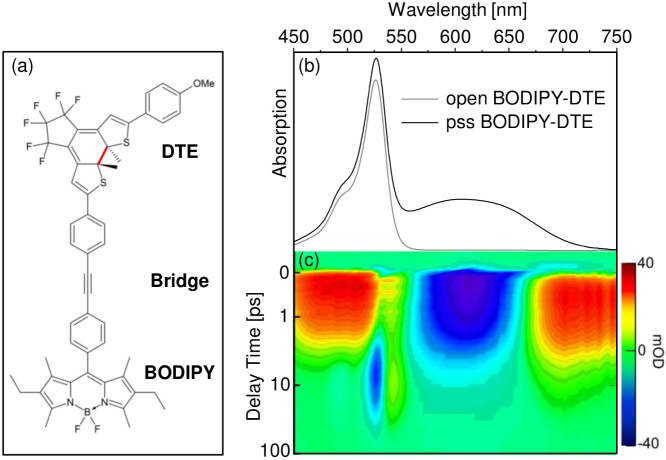
(a) Chemical structure of the closed BODIPY-DTE molecular dyad with photocleavable bond in red; (b) absorption spectra of the open and pss BODIPY-DTE molecular dyad in dichloromethane; (c) transient absorption spectrum after photoexcitation at *λ_pump_* = 600 nm.

The BODIPY-DTE molecular dyad can be efficiently switched between a photostationary state (mixture of closed and open form; pss) and an open form. The pss BODIPY-DTE was prepared by irradiation of the sample containing DTE in its open form with UV light (310 nm). The absorption spectrum of the pss BODIPY-DTE depicted in Fig. 1(b) is composed of a sharp and a broad absorption band at 525 nm and 600 nm which stem from the BODIPY and the DTE moiety, respectively. Time resolved absorption and steady state fluorescence experiments revealed that up to 96% of the DTE is in the closed form (Supplementary Figs. S1 and S2). The spectral characteristics of the BODIPY absorption band is not affected by the switching to the open form with visible light (*λ* > 550 nm), whereas the DTE band at 600 nm completely disappears (cf. Fig. 1(b)).

### Ultrafast spectroscopy

Transient absorption measurements were carried out on the pss BODIPY-DTE. The time resolved spectrum depicted in Fig. 1(c) was recorded after selective photoexcitation of the closed form of the BODIPY-DTE molecular dyad at 600 nm. At delay times < 1 ps characteristic transient absorption signals of closed DTE (cf. Ref. 6) with a negative ground state bleach at *λ_probe_* = 550 nm – 675 nm and positive excited state absorption at *λ_probe_* < 525 nm and > 675 nm are detected. The spectral signature of electronically excited BODIPY (in particular the BODIPY ground state bleach and excited state absorption) is not observed indicating that the electronic excitation does not reach the BODIPY. Time-dependent density functional theory (TDDFT) calculations confirmed that the electronic excitation is strictly located on the DTE and the bridge (Supplementary Fig. S4). The large torsional angle around the single bond connecting the BODIPY to the phenyl ring of the bridge (88° in the ground and 84° in the excited state; from TDDFT calculations) most likely impedes the electronic communication between DTE and BODIPY. In the discussion of the transient absorption data, the DTE and BODIPY are therefore treated as two separated electronic systems, within which only the DTE is in the electronically excited state whereas the BODIPY is in the ground state.

**Figure 2:**
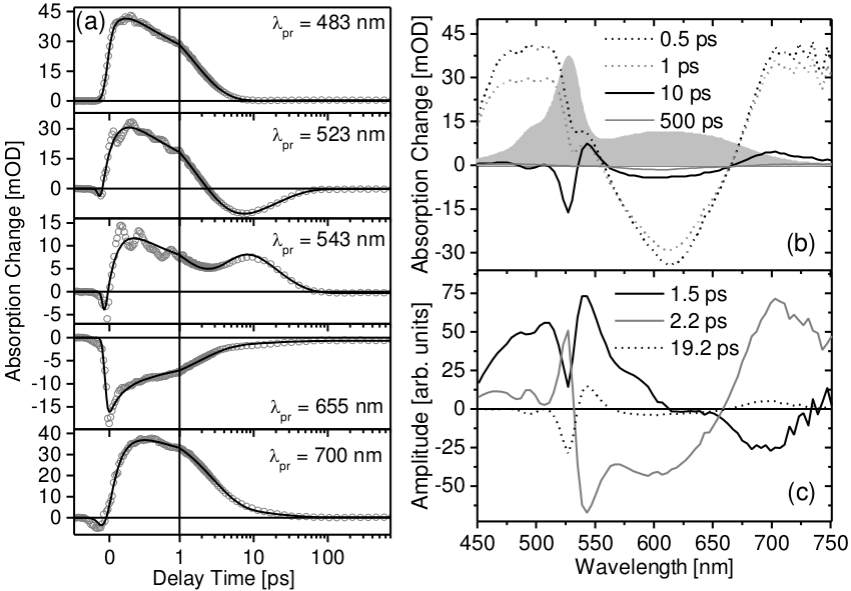
(a) Transient absorption traces recorded at the DTE excited state absorption (*λ_probe_* = 483 nm and 700 nm), the DTE ground state bleach (*λ_probe_* = 655 nm) and in the region of the derivative-like transient absorption signal (*λ_probe_* = 523 nm and 543 nm) after excitation of pss BODIPY-DTE at 600 nm; gray circles and black lines represent the transient absorption data and the fit curves, respectively. (b) Spectra at fixed delay times; gray area represents the pss BODIPY-DTE ground state absorption. (c) Decay associated spectra of *τ*_2_, *τ*_3_ and *τ*_4_ obtained from a global fitting analysis of the transient absorption data.

Interestingly, significant oscillatory patterns are observed at *λ_probe_* = 500 nm – 550 nm within the first picosecond of the transient absorption spectrum in Fig. 1(c). Due to the spectral position these oscillations are most probably related to the absorption band of the BODIPY (cf. absorption spectrum in Fig. 1(b)). At later delay times, spectrally narrow negative and positive difference signals appear at that spectral position. This spectral signature is similar to the derivative of a Gaussian and is therefore termed “derivative-like” in the following. The temporal evolution of both the oscillations and the derivative-like transient absorption signal can be clearly seen in the transient traces at *λ_probe_* = 523 nm and 543 nm in Fig. 2(a).

### DTE relaxation dynamics

Transient traces at the spectral positions of the DTE ground state bleach (*λ_probe_* = 655 nm), the short wavelength excited state absorption (*λ_probe_* = 483 nm) and the long wavelength excited state absorption (*λ_probe_* = 700 nm) are depicted in Fig. 2(a). The long and short wavelength excited state absorptions exhibit different temporal evolutions with a significantly slower decay of the excited state absorption at *λ_probe_* = 700 nm indicating that the bands are related to different electronic transitions. The observed decay kinetics of the long wavelength excited state absorption is comparable to the kinetics of the ground state bleach recovery at *λ_probe_* = 655 nm. The very weak negative offset at *λ_probe_* = 655 nm is due to the conversion of a small fraction of photoexcited DTE to the open form.

The spectra at fixed delay times in Fig. 2(b) show the opposing trends of the transient absorption signals related to photoexcited DTE and the derivative-like signal - the latter increases within the first 10 ps, whereas the DTE ground state bleach and excited state absorption decay strongly.

The complex dynamics after photoexcitation of DTE was evaluated by a global fitting analysis^7^ of the complete set of transient absorption data. Five time constants were necessary to fit the data satisfactorily. For clarity only the decay associated spectra of the time constants *τ*_2_, *τ*_3_ and *τ*_4_ are depicted in Fig. 2(c). The very fast time constant *τ*_1_ (160 fs) is in the range of the temporal resolution of the setup (100 fs) and essentially describes the ultrafast development of the long wavelength excited state absorption whereas *τ*_5_ is necessary to fit the transient absorption signal at maximum delay time. The decay associated spectrum of *τ*_2_ (1.5 ps) in Fig. 2(c) exhibits strong positive amplitudes at the short wavelength excited state absorption and negative amplitudes in the spectral range of the long wavelength excited state absorption. The spectral signature of the ground state bleach cannot be observed. It can be concluded that *τ*_2_ describes a process in the DTE excited state which leads to a decrease of the short wavelength and an increase of the long wavelength excited state absorption. The DTE ground state is not repopulated in that process. The spectrally narrow region of the BODIPY absorption band (*λ_probe_* = 500 nm – 550 nm) is prone to fit artefacts due to the very complex kinetics on a relatively short time scale (c.f. transient trace at *λ_probe_* = 543 nm) which leads to a mirror-image signature of the decay associated spectrum of *τ*_2_ compared to that of *τ*_3_. Consequently, this spectral region is not considered in the discussion of the DTE excited state dynamics. The decay associated spectrum of *τ*_3_ (2.2 ps) contributes with negative amplitudes strongly to the DTE ground state recovery in that spectral region. At the same time positive amplitudes at *λ_probe_* > 650 nm describe the decay of the long wavelength excited state absorption. Consequently, the *τ*_3_-component is related to the relaxation to the DTE electronic ground state.

It is assumed that the transition to the c-DTE and o-DTE ground state occurs via a conical intersection between the S_1_ and the S_0_ potential energy surface^8^. Theoretical studies on cyclohexadiene and dithienylethenes proposed a sequential relaxation from the photoexcited state via a lower lying dark excited state to the electronic ground state. The transitions between the states occur via conical intersections, supposedly driven by the large amplitude of torsional vibrations^9^,^10^. Former time resolved studies on DTE derivatives reported time constants in the range of 0.3 – 60 ps for the photochromic ring opening reaction depending on the substituents^6^,^8^,^11^,^12^. The amplitude pattern in the decay associated spectrum of *τ*_3_ at *λ_probe_* = 500 nm – 550 nm indicates that the electronic relaxation is accompanied by the formation of the derivative-like signal mentioned above. The subsequent decay of that signal is described by the mirror-imaged amplitudes in the decay associated spectrum of *τ*_4_ (19.2 ps). Furthermore, the decay associated spectrum of *τ*_4_ exhibits weak negative amplitudes in the spectral range of the c-DTE absorption and positive amplitudes at *λ_probe_* = 675 nm – 725 nm. Since the DTE excited state decays on a significantly faster time scale (*τ*_3_ = 2.2 ps), these features are most probably not related to the DTE electronic relaxation.

The derivative-like shape at *λ_probe_* = 500 nm – 550 nm indicates that the signal is not a ground state bleach of electronically excited BODIPY but the consequence of a red shifted and/or broadened BODIPY ground state absorption. A transient change in the BODIPY ground state absorption can be explained either by conformational changes of the molecular dyad after relaxation of the DTE to the electronic ground state or by a hot electronic ground state. In former experiments such dynamics have been assigned to cooling in the DTE electronic ground state^6^. If the derivative-like transient absorption signal is related to conformational changes of the molecular dyad, the dynamics should depend on solvent viscosity. Hence, additional transient absorption experiments were conducted on the molecular dyad in a highly viscous solvent. The obtained transient absorption data in Supplementary Fig. S3 demonstrate that only the excited state dynamics is affected by the solvent viscosity, whereas the dynamics of the BODIPY related derivative-like transient absorption signal is essentially identical. Consequently, the observed derivative-like transient absorption signal, which emerges during relaxation of the DTE to the electronic ground state, does most probably not originate from conformational changes but from cooling in the DTE electronic ground state.

### Coherent oscillations

**Figure 3:**
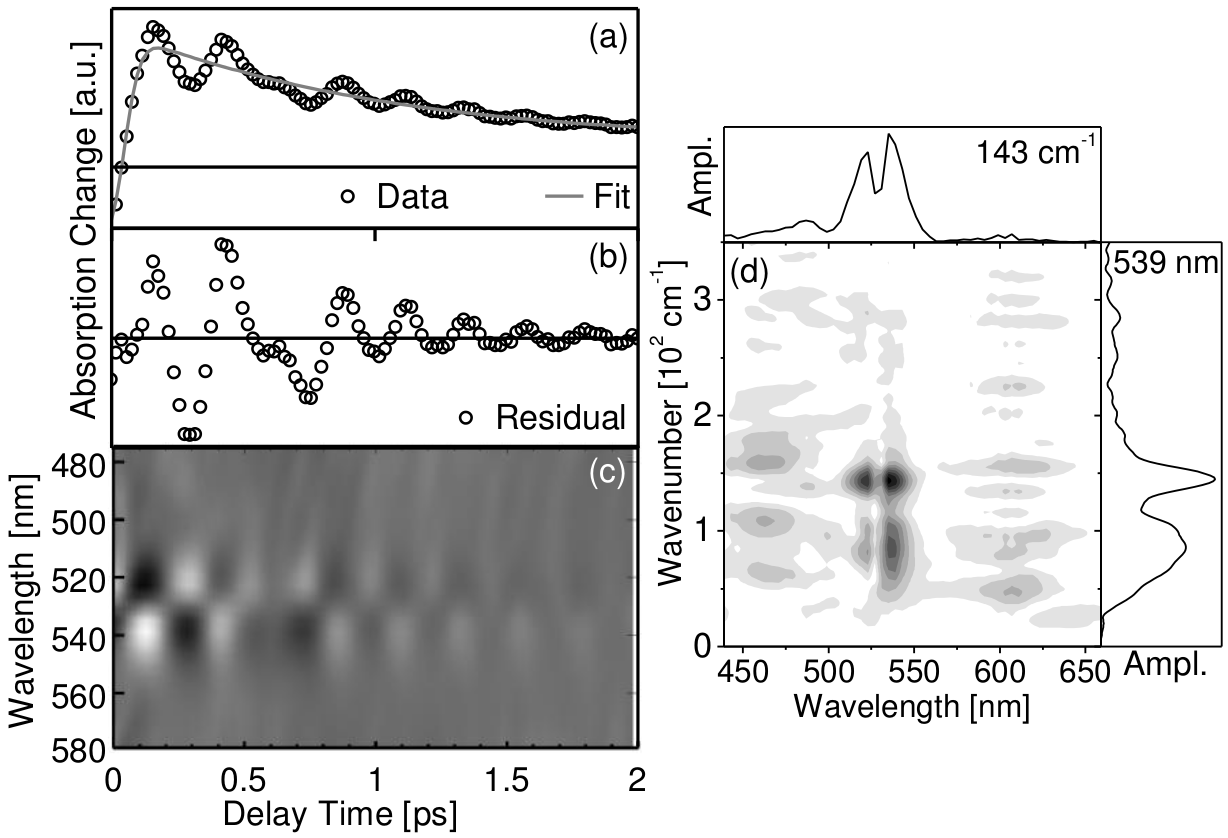
(a) Transient absorption trace recorded at *λ_probe_* = 539 nm after photoexcitation of pss BODIPY-DTE (circles) and corresponding fit (line); (b) residual oscillations obtained by subtraction of the model function; (c) 2D spectrum of the residual oscillations (black and white regions indicate negative and positive values, respectively); (d) Fourier transformed spectrum of the oscillatory residuals.

The observed oscillatory patterns at *λ_probe_* = 500 nm – 550 nm are already present at very early delay times. To identify the origin of these oscillatory signals, the transient absorption data were analyzed via a standard procedure which comprises the fit of the data by a model function consisting of a sum of exponentials convoluted with the instrument response function and a subsequent subtraction (Fig. 3(a) and 3(b))^4^,^13^. The procedure resulted in residuals for each probe wavelength (Fig. 3(c)). Obviously the oscillatory patterns critically depend on the spectral position. A characteristic phase shift of *π* at the maximum of the BODIPY ground state absorption at *λ_probe_* = 530 nm is observed. This clearly indicates that the recorded transient absorption spectrum of pss BODIPY-DTE is frequency modulated (FM)^4^. The FM is due to the presence of coherent nuclear motions of the dyad which causes a modulation of the BODIPY linear absorption spectrum.

The observed oscillations in Fig. 3(b) can be divided into two different time domains: Within the first 0.8 ps the signal is a superposition of two or more modes with a rapid decay of the low frequency contribution. On ultrashort time scales coherent torsional dynamics that drives planarization in the excited state have been observed by Cirmi et al.^1^. After 0.8 ps the oscillations can be attributed to a single vibrational mode. Fourier transformed data of the wavelength dependent residual oscillations are depicted in Fig. 3(d). A Lorentzian-type signal at 143 cm^−1^ and a much broader signal (indicative of a distribution of modes) at lower frequencies, which peaks at 85 cm^−1^, are observed.

### Quantum chemical calculations

To characterize the observed modes, a calculation of the theoretical frequency spectrum was performed at the TDDFT level of theory. In these calculations, the electronic states of the DTE-BODIPY molecular dyad are considered, which feature well-separated excitations on the individual moieties. In particular, the first excited state (S_1_) corresponds to an electronic excitation of the DTE moiety while the BODIPY moiety remains in its ground state. Based on the ground state (S_0_) structure, a geometry optimization in the excited state (S_1_) of the dyad – composed of excited state DTE and ground state BODIPY – was carried out. For this structure, normal mode frequencies were calculated and subsequently corrected by a factor of 0.945 which is the average value of the standard scaling factors for the BLYP density functional and Hartree-Fock (HF) as reported in Ref. 14. In general, these scaling factors have been determined and tested for the electronic ground state. Since scaling factors of the excited states are unknown, we used the well-known factors of the ground state for the excited state.

Since the frequency modulation in the transient absorption experiment indicates a transient shift of the electronic transition energy (ΔE) due to the nuclear motion, we investigate which vibrations give rise to the most significant shift of the transition energy. To this end, the energy differences 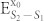 between S_2_ and S_1_ for the relaxed S_1_ structure and the energy differences 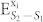 between S_2_ and S_1_ for a displaced structure x*_i_* were calculated. The x*_i_* for the frequency *i* was obtained by applying the normalized displacement vectors 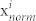 multiplied with a scaling factor a*_i_* on the relaxed S_1_ structure x_0_. The scaling factors a*_i_* were calculated using a harmonic potential so that
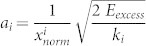
with the excess energy E*_excess_* as the difference between the energy at the Franck-Condon point and the minimum S_1_ energy and k*_i_* as force constant of the normal mode *i*. k*_i_* and 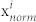 were extracted from the TDDFT calculation. The difference between 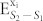 and 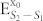 results in the change of the transition energy ΔE*_i_* for each normal mode. ΔE*_i_* values are directly related to Huang-Rhys factors^15^ and should be observable in time resolved experiments as a transient shift of the BODIPY absorption band which modulates the difference signal.

**Figure 4:**
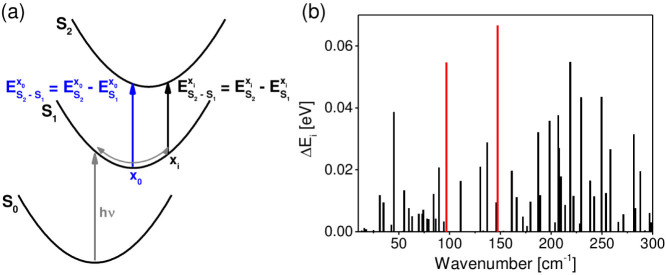
(a) Schematic representation of the displacement induced shift of the electronic excitation energy ΔE*_i_*. (b) Calculated ΔE*_i_* values of the molecular vibrational modes (ΔE*_i_* is calculated as 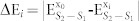).

**Figure 5:**
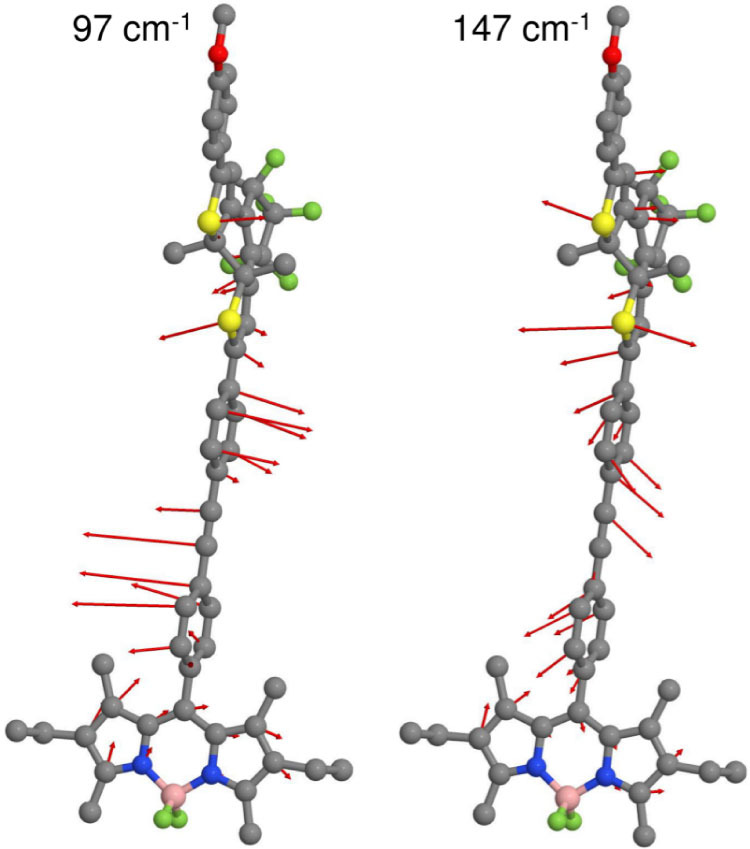
Displacement vectors of the modes at 97 cm^−1^ (left) and 147 cm^−1^ (right) which exhibit a strong shift of the BODIPY-DTE electronic transition energy.

**Figure 6:**
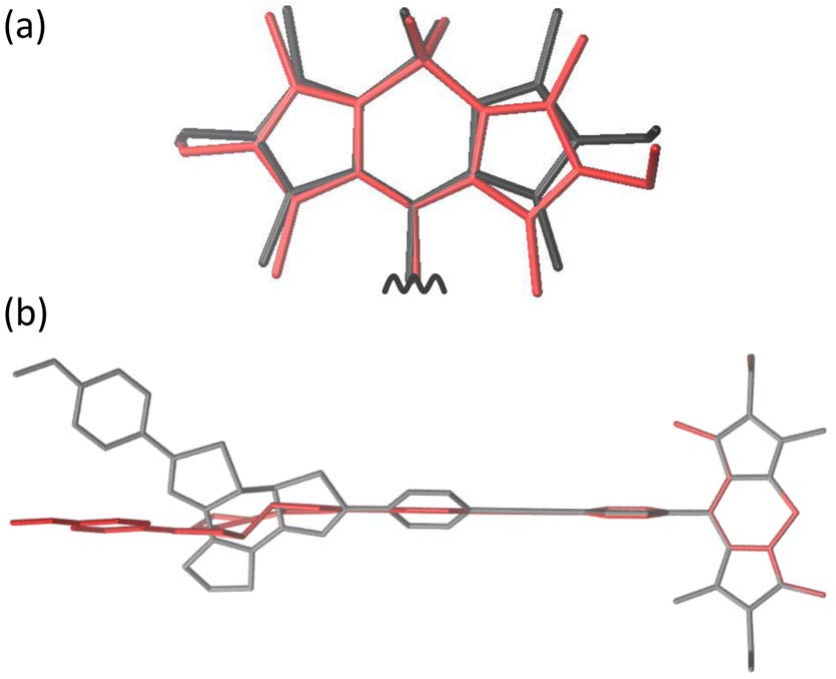
(a) Geometry changes in the BODIPY residue related to the calculated mode at 147 cm^−1^ (outer ring motion). Gray and red color indicate relaxed and displaced structure. (b) Relaxed S_0_ (gray) and the relaxed S_1_ (red) structure of the BODIPY-DTE molecular dyad. For clarity, only the core structure without hydrogens, fluoride and methyl groups at the DTE is shown.

The calculated ΔE*_i_* values for all normal modes of the dyad are shown in Fig. 4(b). The mode with the largest energy shift ΔE*_i_* is found at 147 cm^−1^, in good agreement with the experimentally observed mode at 143 cm^−1^. The corresponding displacement vectors are depicted in Fig. 5. This mode combines geometry changes in the DTE with a pronounced displacement of the bridge (cf. Fig. 5, right; see also Supplementary Movie 1) and a significant motion of the two outer rings of the BODIPY. The outer ring motion is illustrated in Fig. 6(a).

Additional modes with large ΔE*_i_* values are observable at lower wavenumbers, including a distinct frequency at 97 cm^−1^ which combines planarization of the DTE geometry with a displacement of the bridge (cf. Fig. 5, left; see also Supplementary Movie 2). For comparison the relaxed S_0_ and S_1_ structures are depicted in Fig 6(b). The structural changes of the BODIPY moiety are negligible, whereas the changes of the DTE moiety are significant. In contrast to the relaxed S_0_ structure, the connecting bridge and the DTE of the relaxed S_1_ structure are almost planar. Such an excited state planarization is well known and can be directly explained by the reorganization of the single and double bonds of the conjugated *π* system. Consequently, the experimentally observed distribution of low frequency modes can be related to the initial planarization of the DTE. The strong damping of these modes indicates that the planarization occurs on the sub-picosecond time scale.

## Conclusions

In conclusion, the combination of time resolved spectroscopy and TDDFT calculations yields detailed information about the photoinduced processes of a BODIPY-DTE molecular dyad. After selective excitation of DTE, the initial coherent molecular motion out of the initially populated Franck-Condon regime triggers vibrational modes in the electronically isolated BODIPY. The rigid bridge between DTE and BODIPY acts as a molecular piston which transduces the momentum of the DTE geometry change onto the BODIPY. A fast dephasing of low frequency modes is observed which is explained by a fast planarization of the excited state. During the subsequent relaxation process of the DTE the BODIPY molecular probe indicates significant heating of the surrounding medium.

## Methods

### Synthesis of BODIPY-DTE

4,4 Difluoro-8-(4-iodo-phenyl)-2,6-diethyl-1,3,5,7-tetramethyl-4-bora-3a,4a-diaza-s-indacene^16^ and 1-[5-(4-Methoxyphenyl)-2-methylthienyl-3-yl]-2-[2-methyl-5-(4-ethynyl-phenyl)-thien-3-yl]-3,3,4,4,5,5-hexafluorocyclopentene^17^ were synthesized by the literature-known procedures. Coupling of both compounds was achieved according to a method described by Akkaya et al. for related compounds. However, the reaction was carried out at room temperature^18^. The BODIPY-DTE-conjugate was obtained by the reaction of 4,4 Difluoro-8-(4-iodo-phenyl)-2,6-diethyl-1,3,5,7-tetramethyl-4-bora-3a,4a-diaza-s-indacene (37 mg, 0.073 mmol) with 1-[5-(4-Methoxyphenyl)-2-methylthienyl-3-yl]-2-[2-methyl-5-(4-ethynyl-phenyl)-thien-3-yl]-3,3,4,4,5,5-hexafluorocyclo-pentene (50 mg, 0.087 mmol, 1.20 equiv.), [Pd(PPh_3_)_4_] (7 mg, 6 μmol, 8.4 mol%), CuI (0.7 mg, 3 μMol, 4.8 mol%) and triethylamine (abs. 2.5 mL) at room temperature for 19 h (tlc monitoring) in toluene/THF (abs. 1:1, 5 mL) to give after work-up and chromatography (silica gel, hexane/DCM 1:1) the product as an orange-red solid (62 mg, 0.065 mmol, 89%). M.p.: 138°C; Rf = 0.41 (Hexane/CH_2_Cl_2_ 1:1); ^1^H-NMR (400 MHz, CDCl_3_): *δ* = 7.67 (d, ^3^J = 8.08 Hz, 2H), 7.59–7.40 (m, 4H), 7.49–7.45 (m, 2H), 7.34 (s, 1H), 7.30 (d, ^3^J = 8.08 Hz, 2H), 7.17 (s, 1H), 6.94–6.90 (m, 2H), 3.84 (s, 3H), 2.54 (s, 6H), 2.31 (q, ^3^J = 7.48 Hz, 4H), 1.98 (s, 3H), 1.96 (s, 3H), 1.34 (s, 6H), 0.99 (t, ^3^J = 7.54 Hz, 6H) ppm; ^13^C-NMR (100 MHz, CDCl_3_): *δ* = 159.7 (C*_q_*), 154.2 (2C*_q_*), 142.4 (C*_q_*), 142.2 (C*_q_*), 141.4 (C*_q_*), 140.4 (C*_q_*), 139.4 (C*_q_*); 138.4 (2C*_q_*), 136.1 (C*_q_*), 133.5 (C*_q_*), 133.1 (2C*_q_*), 132.4 (4CH*_arom_*), 130.7 (C*_q_*), 128.7 (2CH*_arom_*), 127.1 (2CH*_arom_*), 126.31 (C*_q_*), 126.27 (C*_q_*), 125.8 (C*_q_*), 125.6 (2CH*_arom_*), 123.9 (2C*_q_*), 123.2 (CH*_arom_*), 122.4 (C*_q_*), 121.3 (CH*_arom_*), 114.5 (2CH*_arom_*), 90.5 (C*_q_*), 90.2 (C*_q_*), 55.5 (CH_3_), 17.2 (CH_2_), 14.8 (2CH_3_); 14.7 (2CH_3_), 12.7 (2CH_3_), 12.1 (2CH_3_) ppm; IR (ATR): 3031, 2962, 2928, 2870, 1609, 1540, 1518, 1476, 1439, 1406, 1389, 1374, 1363, 1336, 1321, 1274, 1253, 1192, 1162, 1138, 1115, 1090, 1072, 1054, 1038, 1020, 960, 925, 898, 888, 823, 806, 793, 763, 745, 709, 661 cm^−1^; MS (EI, 260 C): m/z = 952 (100) [M^+^], 937 (34), 476 (10), 262 (10), 142 (12); HR-MS (ESI): calcd for C_53_H_45_BF_8_N_2_OS_2_: 953.3011 [M^+^ + H]; found: 953.2989.

### Sample preparation

A stock solution of the sample in dichloromethane was prepared. For the measurements the stock solution was diluted to a final concentration of approximately 80 μM. This resulted in an optical density of about 0.2 at a wavelength of 600 nm (absorption band of the closed DTE) in a 1 mm cuvette. Before the measurements, the samples were illuminated with UV light of 313 nm using a Hamamatsu LC8 lamp with a filter combination consisting of a UG1 filter (Schott) and a K_2_CrO_4_-solution (700 μmol/L, 1 cm) to reach the photostationary state of the closed isomer. In addition, measurements with solvent of higher viscosity were performed. Therefore, polyethylene glycol (PEG 20.000) was added to the diluted samples and the mixtures were sonicated, resulting in a final PEG concentration of approx. 600 mg/mL.

### Transient spectroscopy

Time resolved experiments were carried out with a conventional transient absorption setup^19^. Short laser pulses (150 fs) of 775 nm at a repetition rate of 1 kHz were provided by a laser-amplifier system (Clark CPA-2110). The probe pulses were generated by focusing the laser fundamental into a sapphire crystal of 2 mm thickness resulting in a single filament white light. For the pump pulses a self-built noncollinear optical parametric amplifier (NOPA)^20^,^21^ was used. It was pumped with the frequency doubled laser fundamental while a white light continuum was used as a seed to generate short laser pulses tunable in the spectral range between 470 nm and 670 nm. The measurements shown in this work were performed at *λ_pump_* = 600 nm and under illumination at 313 nm to prevent the accumulation of a possible photoisomerization product. Considering the absorption features, the pump pulses exclusively photoexcite the closed DTE. This was confirmed in transient absorption measurements on the open form under identical experimental conditions. In these experiments, difference signals could not be detected (data not shown).

### TDDFT calculations

For quantum chemical calculations the BHLYP functional^22^,^23^,^24^,^25^,^26^,^27^ and 6-31G* basis set^28^,^29^ were used within the ab initio program package TURBOMOLE 6.4 (TURBOMOLE V6.4 2012, a development of University of Karlsruhe and Forschungszentrum Karlsruhe GmbH).

## Author Contributions

F.S., L.D., K.R.-B. and Jo.W. initiated this work. M.Z. synthesized the molecule. F.S. and L.D. conducted the experiments. Ja.W. and I.B. contributed to the theoretical study of the dyad. Ja.W. performed the TDDFT calculations. All authors discussed the results and implications at all stages. F.S., L.D., M.B., Ja.W., K.R.-B., I.B. and Jo.W. contributed to the writing of the paper.

## Supplementary Material

Supplementary Information Supplementary Information
