# Supplementary material for: Vibrational coherence transfer in an electronically decoupled molecular dyad
Source: Sci Rep. 2015 Mar 23;5:9368. doi: 10.1038/srep09368 (PMC4369738; doi:10.1038/srep09368)
Supplement: Supplementary Information [file srep09368-s1.pdf]

Supplementary information

Vibrational coherence transfer in an electronically decoupled molecular dyad

F. Schweighöfer[1], L. Dworak[1], M. Braun[1], M. Zastrow[2], J. Wahl[1], I. Burghardt[1], K. Rück-Braun[2], and J. Wachtveitl[1,]*

[1]Institute of Physical and Theoretical Chemistry, Goethe-University Frankfurt, Max-von-Laue Str. 7, D 60438 Frankfurt/M, Germany
[2]TU-Berlin, Straße des 17. Juni 135, D 10623 Berlin, Germany
*wveitl@theochem.uni-frankfurt.de

Steady state measurements

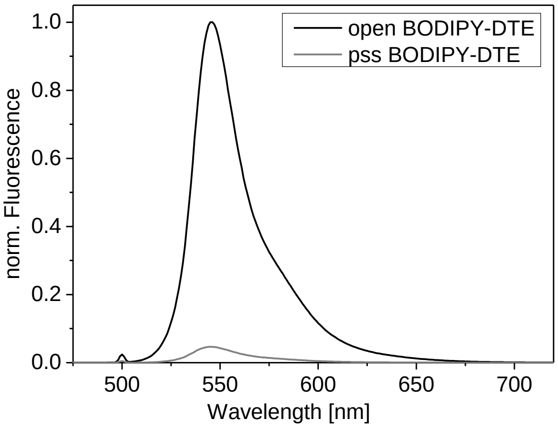

Supplementary Figure S1. Fluorescence spectra of open and pss BODIPY-DTE conjugate; The reversible switching to the pss leads to a quenching of 96 % of the open BODIPY-DTE fluorescence due to an energy transfer from the BODIPY donor to the DTE acceptor. Consequently, at least 96 % of the pss DTE is in the closed form.

Time resolved measurements

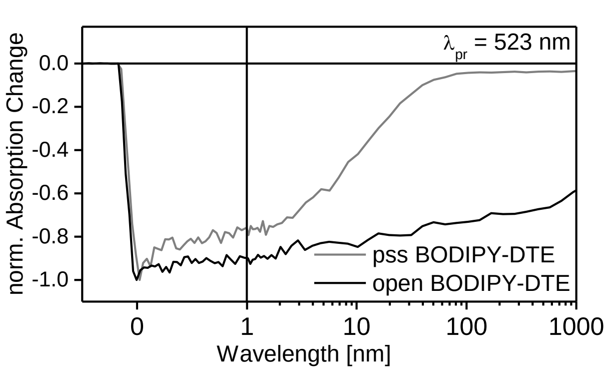

Supplementary Figure S2. Transient absorption traces of open and pss BODIPY-DTE conjugate recorded at the DTE ground state bleach maximum (λ_{pr} = 523 nm) after excitation of the BODIPY moiety at 500 nm. The fast decay of the TA signal in the case of the pss BODIPY-DTE is due to the

energy transfer. On the basis of the residual signals of the two depicted transient traces, a closed DTE content of 94 % in the pss can be estimated.

We fitted single transient traces at both the short and long wavelength side of the excited state absorption signal and compared the results to the ground state bleach decay to confirm the results of the global analysis. The results are depicted in Fig. S3. In all cases a short time constant (0.16 ps) was used to fit the processes on the ultrashort time-scale. The subsequent decay of the excited state absorption at λ_{pr} = 483 nm (short wavelength excited state absorption) can be described very well by a single exponential decay with a time constant of 1.5 ps. In contrast the excited state absorption decay at λ_{pr} = 700 nm is significantly slower and more complex. Three exponentials (1.5 ps, 2.2 ps and 19 ps) are necessary to describe the data satisfactorily. The 1.5 ps-component contributes with negative amplitudes indicating that this time constant describes actually an increase of the positive signal (for amplitudes see Table S1). This increase is overcompensated by the 2.2 ps-component which contributes with positive amplitudes to the signal decay. The decay of the converted signal of the ground state bleach at λ_{pr} = 607 nm is comparable to that at λ_{pr} = 700 nm and can be fitted with three exponentials (2.2 ps, 19 ps and 10 ns as constant offset due to minor switching to the open form). However, the 1.5 ps-component is not necessary to fit the decay dynamics indicating that the ground state is not repopulated with this component. These findings are similar to the global analysis and therefore support the proposed reaction model.

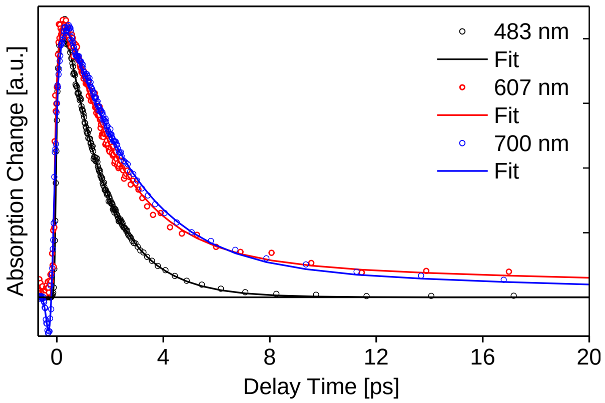

Supplementary Figure S3. Transient absorption traces recorded at the DTE excited state absorption (at 483 nm and 700 nm) and at the DTE ground state bleach maximum (607 nm). For comparison the ground state bleach signal was converted to a positive signal. Transients and fits have been normalized at short delay times.

Table S1. Decay times (τ_x) and amplitudes (a_x) obtained from the fit analysis of the transient traces.

λ_{probe} [nm]	τ_1 [ps]	a_1	τ_2 [ps]	a_2	τ_3 [ps]	a_3	τ_4 [ps]	a_4	τ_5 [ps]	a_5
483	0.16	-0.039	1.5	0.064						
607	0.16	-0.033			2.2	0.044	19	0.0048	10000	0.0012
700	0.16	-0.16	1.5	-0.029	2.2	0.076	19	0.0059		

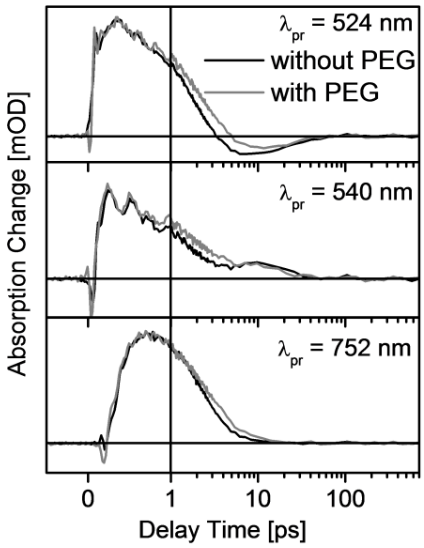

Supplementary Figure S4. Single TA traces recorded for the pss BODIPY-DTE conjugate after photoexcitation of the DTE moiety at 600 nm (black: solvent = DCM, grey: solvent = DCM with PEG approx. 600 mg/mL). After addition of PEG, a slowdown of the relaxation to the electronic ground state can be observed at all probe wavelength showing that the relaxation is accompanied by conformational changes. However the dynamics of the derivative-like signal is not changed, indicating that it most probably cannot be attributed to conformational changes of the molecule in the ground state.

Calculations

For the characterization of the observed modes a calculation of the theoretical frequency spectrum on the TDDFT level of theory has been performed. The calculation has been done using the ab initio program package TURBOMOLE 6.4 [1] in combination with the BHLYP [2, 3, 4, 5, 6, 7, 8] functional and the 6-31G* basis set [9, 10]. As part of these calculations the molecular orbitals of the BODIPY-DTE dyad were calculated. A look on the first two excited states (HOMO to LUMO transition and HOMO-1 to LUMO+1 transition, see fig. S2 and S3) shows that the electronic excitation is localized either on the DTE or on the BODIPY moiety what is consistent with the TA data.

In addition, frequency calculations were performed. Out of these calculations a normal mode with a wavenumber of 147 cm^{-1} can be extracted (see supporting movie). This vibration displaces atoms in the DTE as well as in the BODIPY moiety and thus provides a way to couple both molecular parts mechanically. An outer ring motion of the BODIPY and a strong displacement of the ethinyl bridge are clearly visible in the movie. Considering only the modes with wavenumbers below 300 cm^{-1} (modes with higher frequencies cannot be observed in our TA experiment), the displacement of this vibration (147 cm^{-1}) causes the highest shift of BODIPY excitation energy (ΔE_i) and should therefore be the most visible one.

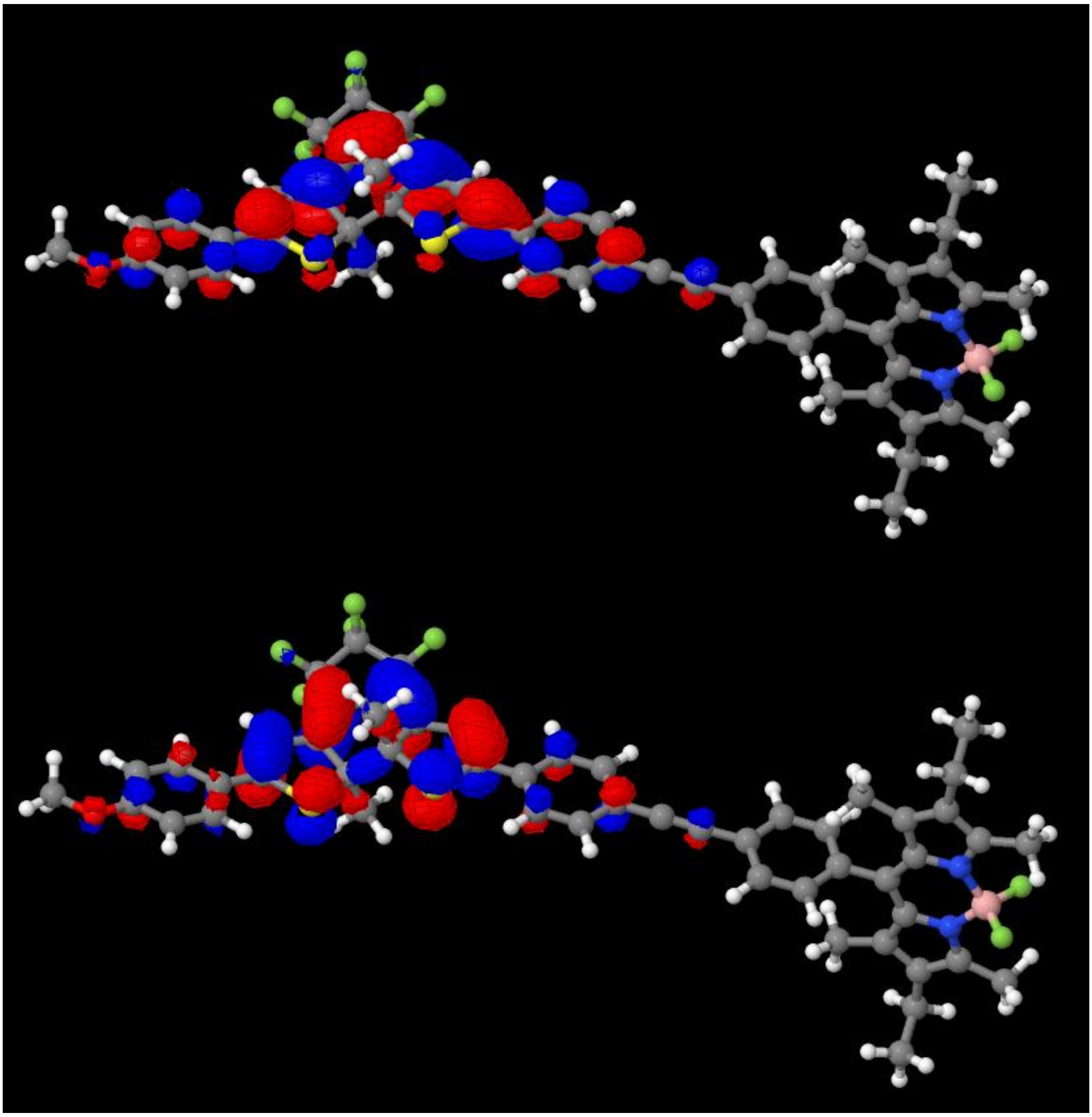

Supplementary Figure S5. First excited state S1 (HOMO (bottom) to LUMO (top) transition) of BODIPY-DTE calculated with TURBOMOLE 6.4. The electronic excitation is strictly localized at the DTE and the bridge.

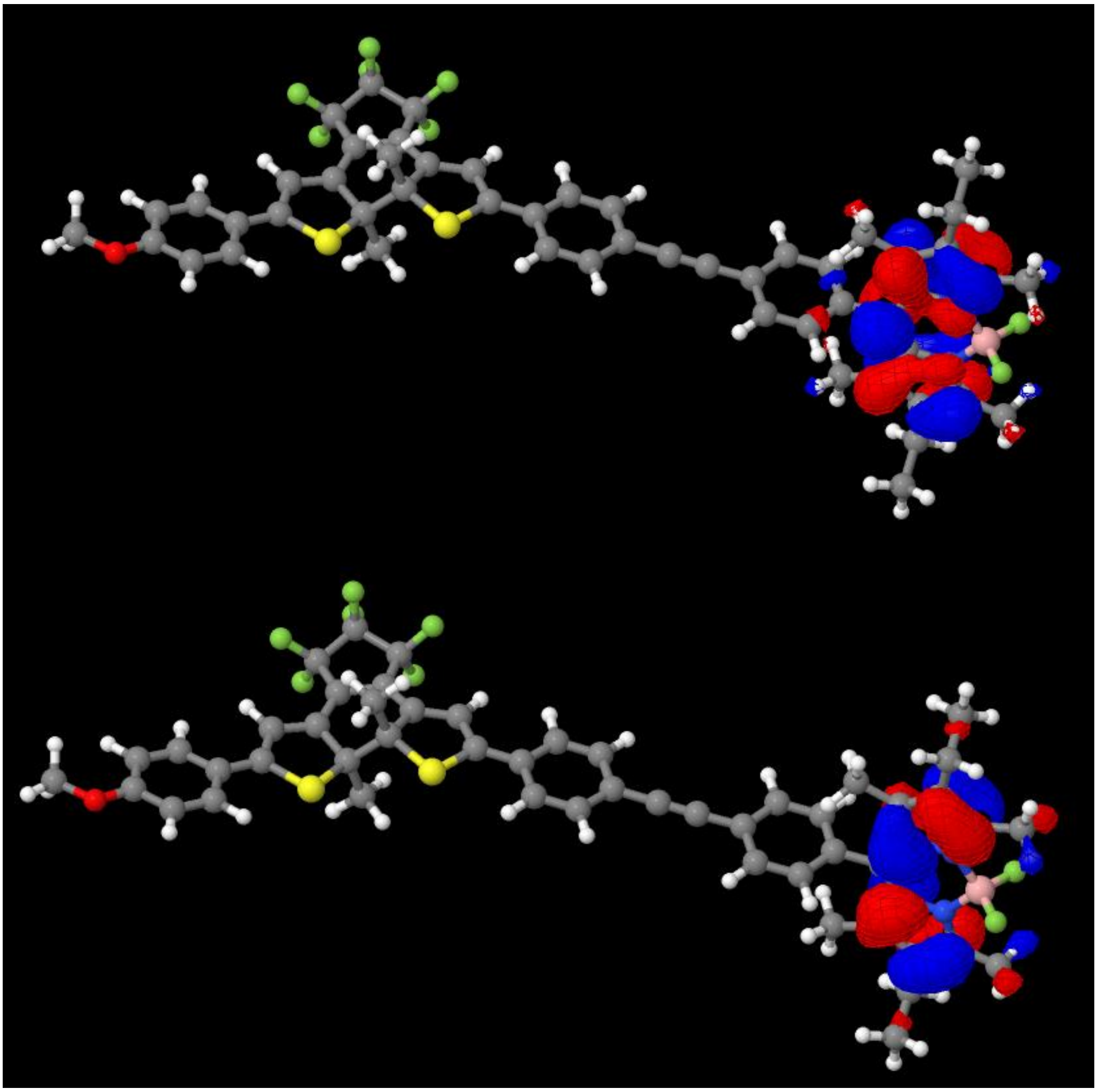

Supplementary Figure S6. Second excited state S2 (HOMO-1 (bottom) to LUMO+1 (top) transition) of BODIPY-DTE calculated with TURBOMOLE 6.4. The electronic excitation is localized at the BODIPY moiety.

References

[1] TURBOMOLE V6.4 2012, a development of University of Karlsruhe and Forschungszentrum Karlsruhe GmbH, 1989-2007, TURBOMOLE GmbH, since 2007; available from http://www.turbomole.com (Date of access: 29/09/2014).

[2] Becke, A. D. Density-functional exchange-energy approximation with correct asymptotic behavior. *Phys. Rev. A* **38,** 3098 (1988) (Date of access: 29/09/2014).

[3] Becke, A. D. A new mixing of hartree-fock and local density functional theories. *J. Chem. Phys.* **98,** 1372 (1993) (Date of access: 29/09/2014).

[4] Dirac, P. Quantum mechanics of many-electron systems. *Proc. R. Soc. Lond. A.* **123,** 714 (1929) (Date of access: 29/09/2014).

[5] Lee, C., Yang, W., Parr, R. G. Development of the colle-salvetti correlation-energy formula into a functional of the electron density. *Phys. Rev. B* **37,** 785 (1988) (Date of access: 29/09/2014).

[6] Slater, J. C. A simplification of the hartree-fock method. *Phys. Rev.* **81,** 385 (1951) (Date of access: 29/09/2014).

[7] Dreuw, A., Weisman, J. L., Head-Gordon, M. Long-range charge-transfer excited states in time-dependent density functional theory require non-local exchange. *J. Chem. Phys.* **119,** 2943 (2003) (Date of access: 29/09/2014).

[8] Pritchard, B., Autschbach, J. Calculation of the vibrationally resolved, circularly polarized luminescence of d-camphorquinone and (s,s)-trans-beta-hydrindanone. *CHEMPHYSCHEM* **11,** 2409 (2010) (Date of access: 29/09/2014).

[9] Binkley, J. S., Pople, J. A., Hehre, W. J. Self-consistent molecular orbital methods. 21. small split-valence basis sets for first-row elements. *J. Am. Chem. Soc.* **102,** 939 (1980) (Date of access: 29/09/2014).

[10] Gordon, M. S., Binkley, J. S., Pople, J. A., Pietro, W. J., Hehre, W. J. Self-consistent molecular-orbital methods. 22. small split-valence basis sets for second-row elements. *J. Am. Chem. Soc.* **104,** 2797 (1982) (Date of access: 29/09/2014).
